# Platelet-derived growth factor (PDGF)-AA/AB in human serum are potential indicators of the proliferative capacity of human synovial mesenchymal stem cells

**DOI:** 10.1186/s13287-015-0239-2

**Published:** 2015-12-10

**Authors:** Mitsuru Mizuno, Hisako Katano, Koji Otabe, Keiichiro Komori, Yukie Matsumoto, Shizuka Fujii, Nobutake Ozeki, Kunikazu Tsuji, Hideyuki Koga, Takeshi Muneta, Akifumi Matsuyama, Ichiro Sekiya

**Affiliations:** Center for Stem Cell and Regenerative Medicine, Tokyo Medical and Dental University, Tokyo, Japan; Department of Cartilage Regeneration, Graduate School, Tokyo Medical and Dental University, Tokyo, Japan; Department of Joint Surgery and Sports Medicine, Graduate School, Tokyo Medical and Dental University, Tokyo, Japan; Department of Bioresources for Drug Discovery, National Institutes of Biomedical Innovation, Health and Nutrition, Osaka, Japan

**Keywords:** Human serum, Mesenchymal stem cell, Synovium, Proliferation, Chondrogenesis

## Abstract

**Introduction:**

For expansion of human mesenchymal stem cells (MSCs), autologous human serum is safer than fetal bovine serum in clinical situations. One of the problems with the use of autologous human serum is that its proliferative effect on MSCs varies widely between donors. The threefold goals of this study were: (1) to demonstrate an improved method for preparing human serum; (2) to identify growth factors predictive of proliferative potential; and (3) to identify a cytokine to promote MSC proliferation in human serum.

**Methods:**

Fresh blood was collected using a closed bag system containing glass beads. The bag was shaken at 20 °C for 30 minutes for rapid preparation, or kept stationary at 4 °C for 24 hours for slow preparation. Passage 0 synovial MSCs derived from four donors were cultured with 10 % conventional rapid preparation serum or modified slow preparation serum from four different donors. To perform the colony-forming unit assay, synovial MSCs were cultured in these serums. The protein expression profile in serum was analyzed using cytokine array. The candidate proteins were speculated from the correlation between the colony-forming ability and protein expression. As an evaluation of the candidate proteins, proliferation ability, surface marker phenotype and differentiation capability of synovial MSCs were examined.

**Results:**

Compared with rapid preparation serum, slow preparation serum resulted in a significantly higher total colony number and twofold higher expression levels of nine proteins (angiopoietin-1, BDNF, EGF, ENA-78, IGFBP-2, platelet-derived growth factor (PDGF)-AA, PDGF-AB/BB, RANTES and TfR). Colony number was positively correlated with PDGF-AA/AB concentrations. Exogenous PDGF-AA significantly promoted proliferation of synovial MSCs, whereas PDGF receptor (PDGFR) inhibitor decreased it. Addition of PDGFs or PDGFR inhibitor did not affect surface epitopes of synovial MSCs. Pretreatment with PDGFs or PDGFR inhibitor did not affect chondrogenic, adipogenic, or calcification potentials of synovial MSCs.

**Conclusion:**

Slow preparation serum contained higher concentrations of PDGF-AA/AB and increased the colony formation number of synovial MSCs. PDGF-AA/AB were indicators of the proliferative potential of human serum. Exogenous PDGF-AA increased proliferation of synovial MSCs without alteration of surface epitopes and differentiation potentials.

## Introduction

Mesenchymal stem cells (MSCs) are used clinically in the field of regenerative medicine [1, 2]. Though MSCs are often expanded with fetal bovine serum (FBS) [3–5], autologous human serum is safer than FBS in clinical situations because it avoids the risk of immune reactions and contamination with pathogens such as prion or zoonotic viruses [6–8]. MSCs derived from synovium have a high chondrogenic potential and synovial MSCs expanded with autologous human serum are used clinically for cartilage regeneration [1, 9–11]. In this setting, autologous human serum is separated after fresh blood is shaken at 20 °C for 30 minutes in a plastic bag containing glass beads [1, 12]. However, this preparation method is not the best. The first objective of this study was to show a better method of preparing human serum with more proliferative potential.

One of the problems with the use of autologous human serum is that its proliferative effect on MSCs varies widely between donors [13]. Human serum with inferior quality may result in too low a yield of MSCs to be transplanted in clinical situations. To obtain a sufficient number of MSCs with absolute certainty, it is essential to predict the proliferative potential of human serum. In addition, since serum contains a great variety of growth factors, attention should be paid to those that are desirable. The second objective of this study was to identify growth factors that predict the proliferative potential of synovial MSCs expanded in human serum.

When inferior quality of human serum is a concern, rescuing the serum is required in clinical situations. One possibility is to add a growth factor to the serum, in which case this growth factor should not affect MSC properties. The third objective of this study was to identify a cytokine to promote proliferation of synovial MSCs cultured by human serum.

## Methods

### Collection of human serum

**Fig. 1:**
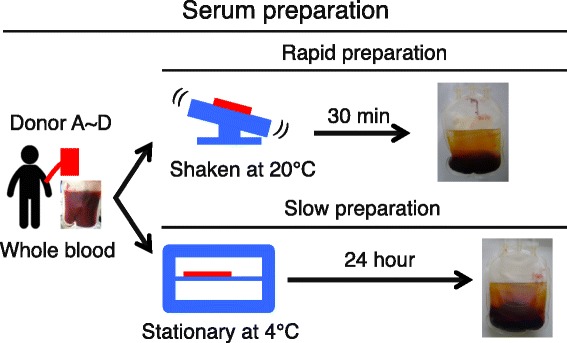
Schema of rapid preparation and slow preparation for sera

The present study was approved by the Medical Research Ethics Committee of Tokyo Medical and Dental University (approved No. 1431) and all human study subjects provided informed consent. Fresh blood was collected from four healthy male volunteers (32–36 years) using a closed bag system (JMS Co., Ltd, Hiroshima, Japan) [14]. The bag containing glass beads was shaken at 20 °C for 30 minutes for rapid preparation, or kept stationary at 4 °C for 24 hours for slow preparation, and then serum in each group was separated (Fig. 1). These sera was filtered through a 0.45-μm nylon filter (Thermo Fisher Scientific, Inc., Waltham, MA, USA) and stored at –20 °C until use.

### Quantification of cytokine expression levels

The relative levels of cytokines in sera were analyzed using a Human XL Cytokine Array (R&D Systems, MN, USA) according to the manufacturer’s instructions. The positive spots were semi-quantitatively evaluated by the Image Lab software (Bio-Rad Laboratories, Richmond, CA, USA). The serum levels of platelet-derived growth factor (PDGF)-AA, PDGF-AB and PDGF-BB were measured by sandwich enzyme-linked immunosorbent assay (ELISA; DAA00B, DHD00C and DBB00; R&D Systems) following the manufacturer’s protocol.

### Synovial MSCs

Human synovium was harvested from the knees of seven donors (71–83 years) with osteoarthritis during total knee arthroplasty. Synovium was digested in a solution of 3 mg/mL collagenase (Sigma-Aldrich Japan, Tokyo, Japan) in Hanks’ balanced salt solution (HBSS; Gibco, Waltham, MA, USA) at 37 °C. After 3 hours, the digested cells were filtered through a 70-μm cell strainer (Greiner Bio-One GmbH, Frickenhausen, Germany). The cells were cultured in human serum culture medium, α-minimum essential medium (α-MEM; Gibco) supplemented with 1 % antibiotic-antimycotic (Gibco) and with 10 % human serum without heat inactivation in a cell culture incubator (Astec, Fukuoka, Japan) set 37 °C and 5 % CO_2_. The cells were counted with a disposable cell counting plate (One Cell Inc., Shiga, Japan) to determine the number of cells.

### Colony formation and proliferation of synovial MSCs

For colony formation assay, passage 0 synovial MSCs from four donors were plated on six-well plates at 100 cells/10 cm^2^ well and cultured with 10 % rapid or slow preparation serum from four other donors. The well was stained with crystal violet at 14 days and the colony number was counted. For proliferation assay, passage 1 synovial MSCs from three donors were plated on six-well plates at 100 cells/cm^2^ well and cultured with 10 % rapid preparation serum from one other donor. The cells were harvested with 0.25 % trypsin and 1 mM EDTA (Gibco) at 37 °C for 5 minutes and counted with a cell-counting plate.

### Supplementation of growth factors

The culture medium, α-MEM containing 10 % human rapid preparation serum, was supplemented with 5 ng/mL PDGF-AA, PDGF-AB, PDGF-BB (R&D Systems), 10 nM Crenolanib as a PDGF receptor (PDGFR) α and β inhibitor (MedChem Express, NJ, USA) or vehicle (dimethyl sulfoxide; Wako, Tokyo, Japan) as a control.

### Flow cytometric analysis

Cultured synovial MSCs from three donors at passage 1 were harvested using a cell-dissociation buffer. Cells were suspended in HBSS at a density of 5 × 10^5^ cells/mL and stained for 30 minutes on ice with the antibodies CD31-PE-Cy7 (Becton, Dickinson and Company; BD, Franklin Lakes, NJ, USA), CD45-APC-H7 (Biolegend, San Diego, CA, USA), CD44-APC-H7 (BD), CD73-BV421 (BD), CD90-PE (BD), CD105-PerCP-Cy5.5 (BD), CD140a-BV421 (BD), CD140b-PerCP-Cy5.5 (BD), CD146-FITC (BD) and CD271-APC (Miltenyi Biotec) for cell surface analysis. Flow cytometric analysis of the cell surface was performed by a triple-laser FACS Verse™ system (BD).

### Differentiation assay

Cultured synovial MSCs from three donors at passage 1 were harvested using 0.25 % trypsin and 1 mM EDTA.

For adipogenic differentiation, adherent cells were cultured in adipogenic induction medium (Lonza, Basel, Switzerland), which was changed every 3–4 days. After 21 days, oil red-o staining (Muto Pure Chemicals, Tokyo, Japan) confirmed the differentiation of these cells into adipocytes [15]. To quantify the amount of oil red-o, the stained oil droplets were extracted by 2-propanol (Wako, Tokyo, Japan) and oil red-o stain in extraction buffer was determined by measuring the optical density of the solution at 510 nm [16].

For osteogenic differentiation, 100 cells were transferred to a 100-mm dish and cultured for 14 days in culture medium. Adherent cells were cultured in osteogenic induction medium containing 50 μg/mL ascorbic acid 2-phosphate (Wako), 10 nM dexamethasone (Wako) and 10 mM β-glycerophosphate (Sigma-Aldrich), which was changed every 3–4 days. After 21 days, the differentiation of these cells into osteoblasts was assessed by alizarin red staining (Merck Millipore, Billerica, MA, USA) [17]. To quantify the amount of alizarin red, the deposition was extracted by 10 % (w/v) cetylpyridinium chloride (Sigma-Aldrich) in 10 mM sodium phosphate (pH 7.0) at room temperature for 1 hour and the alizarin red stain in extraction buffer was determined by measuring the optical density of the solution at 560 nm [16].

For chondrogenic differentiation, 2.5 × 10^5^ cells were transferred to a 15-mL tube (BD Falcon) and cultured in chondrogenic induction medium containing 10 ng/mL transforming growth factor-β3 (Miltenyi Biotec Japan, Tokyo, Japan) and 500 ng/mL bone morphogenetic protein 2 (Medtronic), which was changed every 3–4 days. After 21 days, chondrogenic differentiated cells were analyzed by toluidine blue (Wako) staining.

### Statistical analysis

All data were statistically evaluated by analysis of variance with GraphPad Prism 6 (GraphPad Software, La Jolla, CA, USA). Data are expressed as mean ± SD. Each statistical analysis method is described in the legend. Two-tailed *P* values < 0.05 were considered to be significant.

## Results

### Colony number of synovial MSCs by rapid and slow preparation serum

**Fig. 2:**
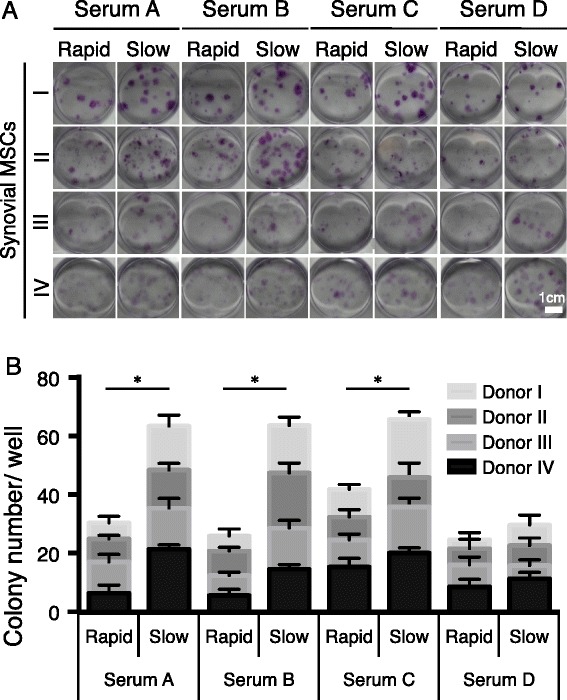
Colony number of synovial mesenchymal stem cells (*MSCs*) by rapid and slow preparation serum. **a** Representative wells stained with crystal violet. Sera derived from four donors and synovial MSCs derived from other four donors were used. **b** Quantification of colony number. Data are shown as mean ± SD (n = 6 in a minimum unit). *p < 0.001 by two-way analysis of variance with repeated measures

Synovial MSCs formed cell colonies 14 days after culture with conventional rapid preparation serum (Fig. 2a). The use of slow preparation serum, a modified method, resulted in a significantly higher number of cell colonies in three of four donors (Fig. 2b). The colony number obtained by rapid preparation serum was 7.4 ± 3.7 and was 14.5 ± 5.5 by slow preparation serum on average when sera from four donors and synovial MSCs derived from another four donors were used.

### Cytokine expression levels in rapid and slow preparation serum

**Fig. 3:**
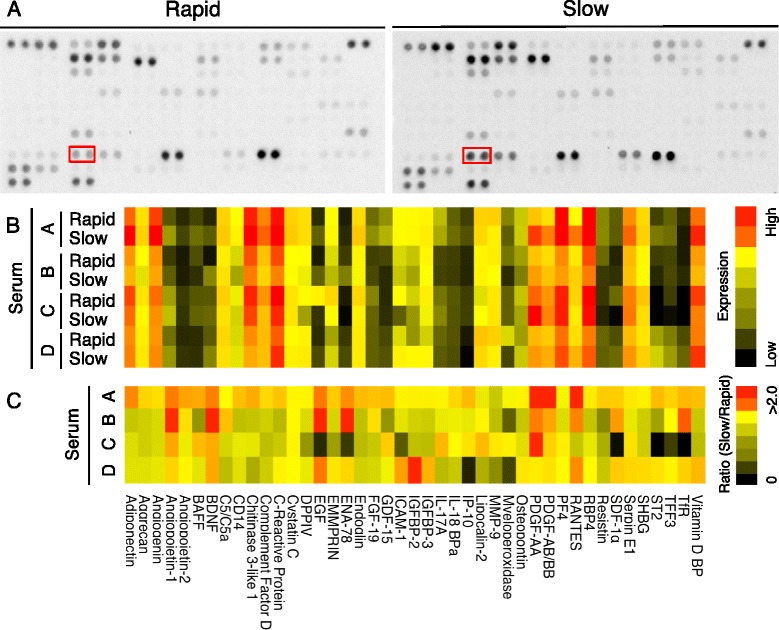
Cytokine expressions in rapid and slow preparation serum. **a** Representative images of cytokine arrays. Each antibody is spotted in duplicate on a nitrocellulose membrane. Spots for PDGF-AA are surrounded with red squares. **b** Heat map for cytokine expression level in rapid and slow preparation sera in four donors. **c** Heat map for ratio of cytokine expression level in slow preparation serum to cytokine expression level in rapid preparation serum. See Table 1 for a list of proteins

We analyzed eight serum samples from four donors by cytokine arrays and detected 43 positive spots among 102 proteins examined (Fig. 3a, Table 1). A heat map showing the pixel density of these spots demonstrated that, for most cytokines detected, expression levels depended on serum preparation methods (Fig. 3b). According to a heat map for the ratio of each protein expression in slow preparation serum to that in rapid preparation serum, the ratio was twice as high in nine cytokines (angiopoietin-1, BDNF, EGF, ENA-78, IGFBP-2, PDGF-AA, PDGF-AB/BB, RANTES and TfR) at least in one donor per protein (Fig. 3c).

**Table 1:** Proteins that could be visualized by cytokine arrays for human serum

Adiponectin	IGFBP-3
Aggrecan	Interleukin (IL)-17A
Angiogenin	IL-18 BPa
Angiopoietin-1	IP-10; CXCL10
Angiopoietin-2	Lipocalin-2
BAFF; B-cell activating factor belonging to the tumor necrosis factor family	MMP-9; matrix metalloproteinase-9
BDNF; brain-derived neurotrophic factor	Myeloperoxidase
Complement component C5/C5a	Osteopontin
CD14	Platelet-derived growth factor (PDGF)-AA
Chitinase 3-like 1	PDGF-AB/BB
Complement factor D	PF4; CXCL4
CRP; C-reactive protein	RANTES; regulated on activation, normal T cell expressed and secreted, CCL5
Cystatin C	RBP4; retinol-binding protein 4
DPPIV; dipeptidyl-peptidase IV, CD26	Resistin; adipose tissue-specific secretory factor (ADSF)
EGF; epidermal growth factor	SDF-1α; stromal cell-derived factor 1, CXCL12
EMMPRIN; CD147, extracellular matrixm metalloproteinase inducer	Serpin E1
ENA-78; epithelial-derived neutrophil-activating peptide 78, CXCL5	SHBG; sex hormone-binding globulin
Endoglin; CD105	ST2; IL1RL1 (interleukin 1 receptor-like 1)
FGF-19; fibroblast growth factor-19	TFF3; trefoil factor 3
GDF-15; growth differentiation factor 15 MIC-1 (macrophage inhibitory cytokine-1)	TfR; transferrin receptor 1 (CD71)
ICAM-1; CD54	Vitamin D BP
IGFBP-2; insulin-like growth factor-binding protein 2	

### Correlation between colony number of MSCs and PDGF concentrations in sera

**Fig. 4:**
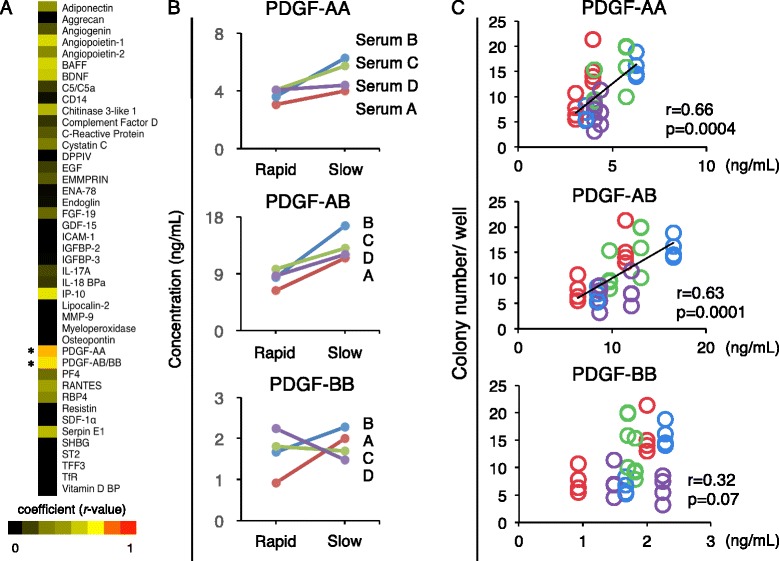
Correlation between colony number of synovial MSCs and platelet-derived growth factor (*PDGF*) concentrations in sera. **a** Heat map for *r*-value in correlation analysis between cytokine expression levels measured by cytokine array and colony formation number of synovial MSCs. *p < 0.05 and *r* > 0.5 by Pearson correlation coefficient. **b** Concentration of PDGF isoforms in serum measured by ELISA. **c** Correlation analysis between concentrations of PDGF isoforms in sera and colony formation numbers of synovial MSCs. See Table 1 for a list of proteins

A heat map of *r*-values, obtained after correlation analysis between cytokine expression levels and colony formation numbers, revealed that *r*-values were higher than 0.5 for PDGF-AA and AB/BB (Fig. 4a). PDGF-AA and PDGF-AB concentrations measured by ELISA were higher in slow preparation serum than in rapid preparation serum in all four donors, while PDGF-BB concentration measured by ELISA was not (Fig. 4b). There were positive correlations between colony numbers and concentrations for both PDGF-AA (*r* = 0.66) and PDGF-AB (*r* = 0.63) (Fig. 4c).

### Effect of exogenous PDGFs on proliferation of synovial MSCs

**Fig. 5:**
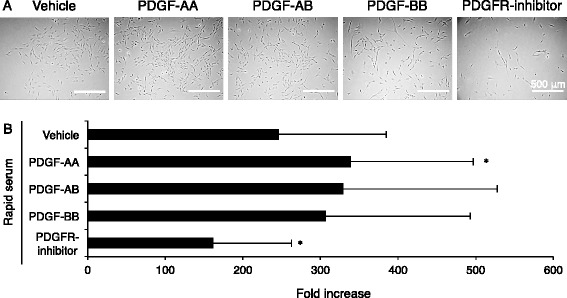
Effect of exogenous platelet-derived growth factors (*PDGFs*) on proliferation of synovial MSCs. **a** Representative cell morphology. Synovial MSCs were plated at 100 cells/ cm^2^ and cultured for 10 days in the presence of PDGF isoforms or PDGF receptor (*PDGFR*) inhibitor. **b** Fold increase of synovial MSCs. Data are shown as mean ± SD (n = 9). *p < 0.05, versus rapid preparation with vehicle by one-way analysis of variance followed by Dunnett's multiple comparisons

To examine the effect of PDGFs on proliferation of synovial MSCs, they were cultured with PDGF-AA, -AB, -BB, Crenolanib (PDGFR α and β inhibitor) or vehicle (dimethyl sulfoxide). PDGFs and PDGFR inhibitor did not affect the morphology of synovial MSCs (Fig. 5a). PDGF-AA significantly increased and PDGFR inhibitor significantly decreased the proliferation of synovial MSCs (Fig. 5b).

### Effect of exogenous PDGFs on surface markers of synovial MSCs

**Fig. 6:**
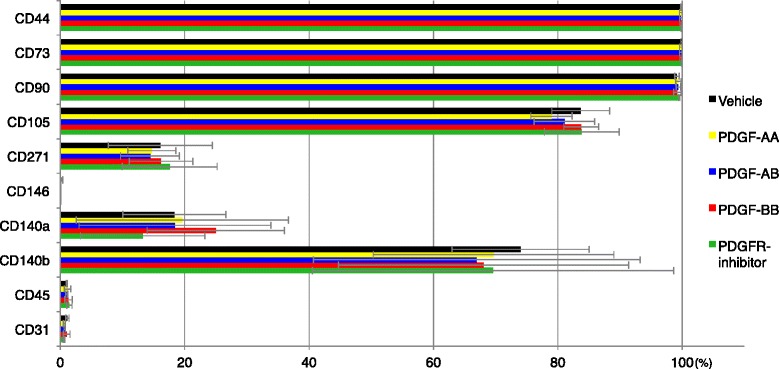
Effect of exogenous platelet-derived growth factors (*PDGFs*) on surface markers of synovial MSCs. Synovial MSCs derived from three donors were plated at 100 cells/cm^2^ and cultured for 10 days in the presence of PDGF isoforms and PDGF receptor (*PDGFR*) inhibitor. Data are shown as mean ± SD

Synovial MSCs expressed CD44, CD73 and CD90 at high rates (over 90 %), CD105 and CD140b at a moderate rate (over 50 %), CD140a and CD271 at low rates (approximately 20 %), and did not express CD31, CD45 and CD146. Exogenous PDGFs and PDGFR inhibitor did not affect these surface markers (Fig. 6).

### Effect of exogenous PDGFs on differentiation potentials of synovial MSCs

**Fig. 7:**
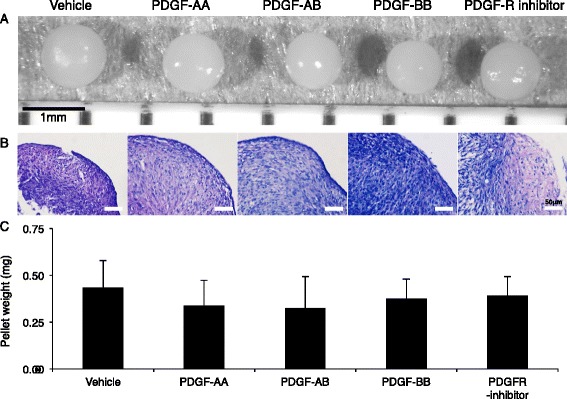
Effect of pretreatment of platelet-derived growth factors (*PDGFs*) on chondrogenesis of synovial MSCs. **a** Representative macroscopic appearance. Synovial MSCs derived from three donors were pretreated with PDGFs, and then differentiated into cartilage pellets (n = 6 in each donor). **b** Histological sections stained with toluidine blue. **c** Wet weight of pellets. Data are shown as mean ± SD (n = 18). *PDGFR* PDGF receptor

Synovial MSCs were pretreated with PDGFs and PDGFR inhibitor, and then differentiated into cartilage. Irrespective of exogenous PDGFs, cartilage pellets were spherical (Fig. 7a), and the matrix showed a purple color after toluidine blue staining (Fig. 7b). Pretreatment with PDGFs did not affect pellet weight (Fig. 7c), an indicator of chondrogenic potential.

**Fig. 8:**
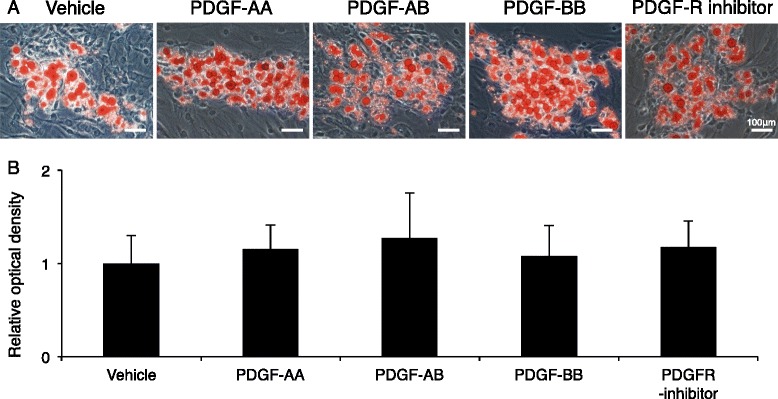
Effect of pretreatment of platelet-derived growth factors (*PDGFs*) on adipogenesis of synovial MSCs. **a** Representative cell morphology stained with oil red-o. Synovial MSCs were pretreated with PDGFs, and then differentiated into adipocytes. **b** Relative optical density for oil red-o. Data are shown as mean ± SD (n = 4). *PDGFR* PDGF receptor

After adipogenic induction, synovial MSCs contained lipid shown as red after oil red-o staining irrespective of exogenous PDGFs (Fig. 8a). Pretreatment with PDGFs did not affect adipogenic potential evaluated by optical density for oil red-o (Fig. 8b).

**Fig. 9:**
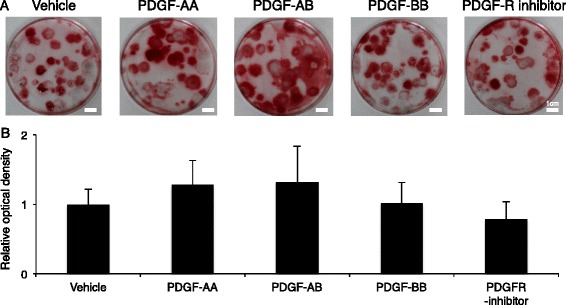
Effect of pretreatment of platelet-derived growth factors (*PDGFs*) on calcification of synovial MSCs. **a** Representative culture dishes stained with alizarin red. Synovial MSCs were pretreated with PDGFs, and then calcified. **b** Relative optical density for alizarin red. Data are shown as mean ± SD (n = 6). *PDGFR* PDGF receptor

After calcification induction, synovial MSCs formed alizarin red positive colonies irrespective of exogenous PDGFs (Fig. 9a). Pretreatment with PDGFs did not affect calcification potential evaluated by optical density for alizarin red (Fig. 9b).

## Discussion

In this study we showed that slow preparation serum formed more cell colonies than rapid preparation serum. Fresh blood was collected in a closed bag containing glass beads. The bag was shaken at 20 °C for 30 minutes for rapid preparation, or kept stationary at 4 °C for 24 hours for slow preparation. These stationary conditions have been shown to promote the release of growth factors from platelets, such as PDGFs [18, 19]. Proper management of this human serum isolation protocol would be able to comply with good gene, cellular and tissue-based products manufacturing practice (GCTP) for clinical or commercial use, since this method is very simple.

Low stability of human serum may cause growth failure of MSCs and discontinuation of clinical cell transplantation. In the present study, by analyzing the relationships between serum protein profiles and colony formation of synovial MSCs, we demonstrated that PDGF-AA and PDGF-AB were the growth factors predictive of the proliferative potential of human serum. We also showed that supplementation with PDGF-AA improved the proliferation of MSCs. If the concentration of PDGF-AA in serum is low, the addition of PDGF-AA will compensate for the quality of the serum. The intractable problems caused by individual differences in human serum may thus be resolved.

Exogenous PDGFs did not affect MSC-related surface marker expressions. Positive rate for CD140a (PDGFRα) was around 20 % and this was lower than that in our previous reports [12]. Our recent experiments indicated that the positive rate for CD140a decreased by trypsin (manuscript in preparation). In our current study, treatment with trypsin might affect CD140a expression of human synovial MSCs.

Pretreatment with PDGFs did not affect chondrogenic potential of synovial MSCs, though PDGF signaling was significant in chondrogenesis [20]. Pretreatment with PDGFs also did not affect adipogenic and calcification potential of synovial MSCs. This is possibly because human serum includes various growth factors that help to compensate differentiation ability. Therefore, it is estimated that PDGF supplementation would not adversely affect outcomes after transplantation in vivo. This is the most important point for regenerative medicine in clinical situations that place great value on safety [21]. The concentrations of PDGFs needed to examine the effect of exogenous PDGFs on synovial MSCs were set up to be similar to human serum.

We propose three limitations in this study. First, we did not examine the proliferative effect of cells other than synovial MSCs, though the effects of PDGF-AA and -AB can be expected in MSCs derived from other tissues, such as bone marrow and adipose tissue, based on their PDGFR expression [22, 23]. Second, we focused on 43 cytokines detected with a cytokine array kit that is designed to determine the selected 102 cytokines but did not examine the effect of other cytokines not included in this kit. Indeed, synovial MSCs cultured with PDGFR inhibitor still increased, suggesting that other cytokines such as EGF [24] compensated for proliferative effects. Third, human serum derived from only one donor was used to examine the effects of exogenous PDGFs. Donor variety of responsiveness to PDGFs may not be ignored.

## Conclusions

Slow preparation serum contained higher concentrations of PDGF-AA/AB and increased the colony formation number of synovial MSCs. PDGF-AA/AB were indicators of the proliferative potential of human serum. The addition of PDGF-AA increased proliferation of synovial MSCs without alteration of surface epitopes and differentiation potentials.

## Abbreviations

α-MEM: Alpha minimum essential medium
BDNF: Brain-derived neurotrophic factor
EGF: Epidermal growth factor
ELISA: Enzyme-linked immunosorbent assay
ENA-78: Epithelial-derived neutrophil-activating peptide 78
FACS: Fluorescence-activated cell sorter
FBS: Fetal bovine serum
HBSS: Hanks’ balanced salt solution
IGFBP-2: Insulin-like growth factor-binding protein 2
MSC: Mesenchymal stem cell
PDGF: Platelet-derived growth factor
PDGFR: Platelet-derived growth factor receptor
RANTES: Regulated on activation, normal T cell expressed and secreted
TfR: Transferrin receptor 1
